# Soil Contamination by a Lead Smelter in Brazil in the View of the Local Residents

**DOI:** 10.3390/ijerph15102166

**Published:** 2018-10-02

**Authors:** Fernando M. Carvalho, Tania M. Tavares, Liliane Lins

**Affiliations:** Health, Environment, and Work Program, School of Medicine, Federal University of Bahia. Largo do Terreiro de Jesus, s/n, Centro Histórico, Salvador, Bahia 40026-010, Brazil; ttavares@ufba.br (T.M.T.); liliane.lins@ufba.br (L.L.)

**Keywords:** soil pollution, lead, public perception of science, environmental hazards, solid waste, toxic wastes

## Abstract

A primary lead smelter operated in Santo Amaro City in Brazil from 1960 to 1993, leaving approximately 500,000 tons of industrial dross containing 2–3% of lead and other toxic elements that contaminated the industry grounds and the urban environment. This study aimed to present the local residents’ perception towards soil contamination by the smelter. In a cross-sectional study, 208 residents from randomly selected households were interviewed about dross hazards and proposals for its management. A city map depicts the distribution and concentration of lead, cadmium, arsenic, zinc, nickel, and antimony, measured in the soil of the 39 households with visible smelter dross. Only one site complies with the soil quality reference values; 27 (69.2%) call for preventive measures, and 11 (28.2%) require intervention. The smelter dross continues widely spread over the city. Thirty (76.9%) out of the 39 residents were able to recognize the smelter dross on household surroundings. However, this ability was not associated with the concentrations of toxic elements in the soil of their residences and surroundings. The smelter and the local Prefecture were most frequently held liable for taking soil cleanup actions. The most frequently (38.0%) cited solution for managing the dross found in the households was “to provide the residents with information about health risks related to the dross”.

## 1. Introduction

**Figure 1:**
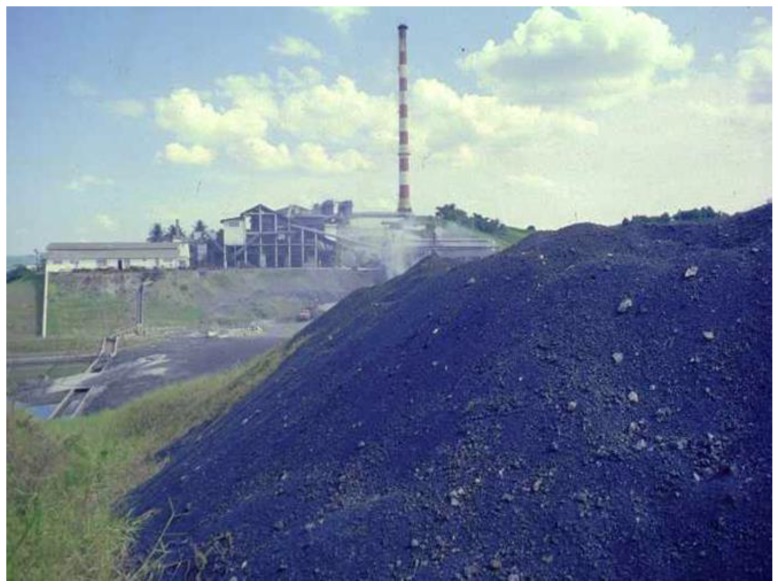
The big dross pile on the smelter ground, 1999.

**Figure 2:**
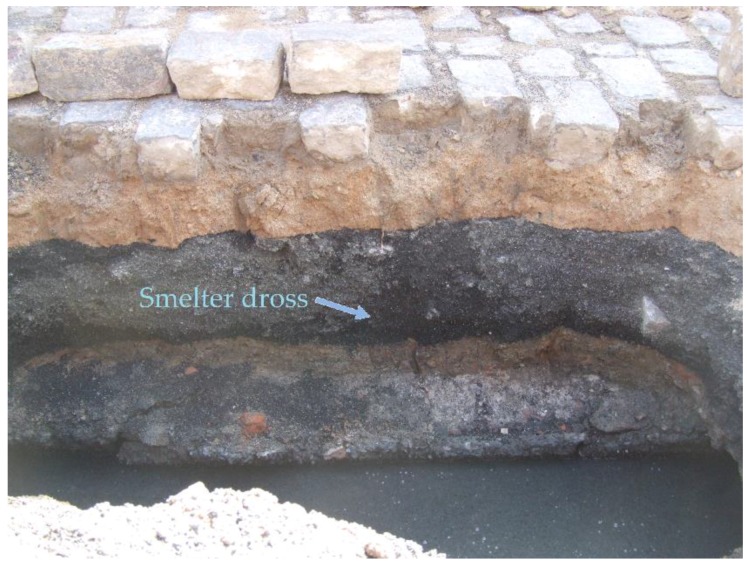
The smelter dross layer under the street pavement, 2008.

**Figure 3:**
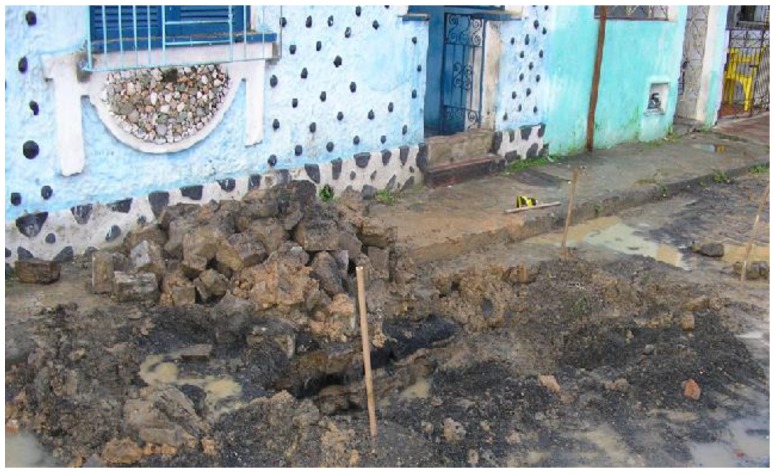
The dross exposed during water pipe repair, 2005.

**Figure 4:**
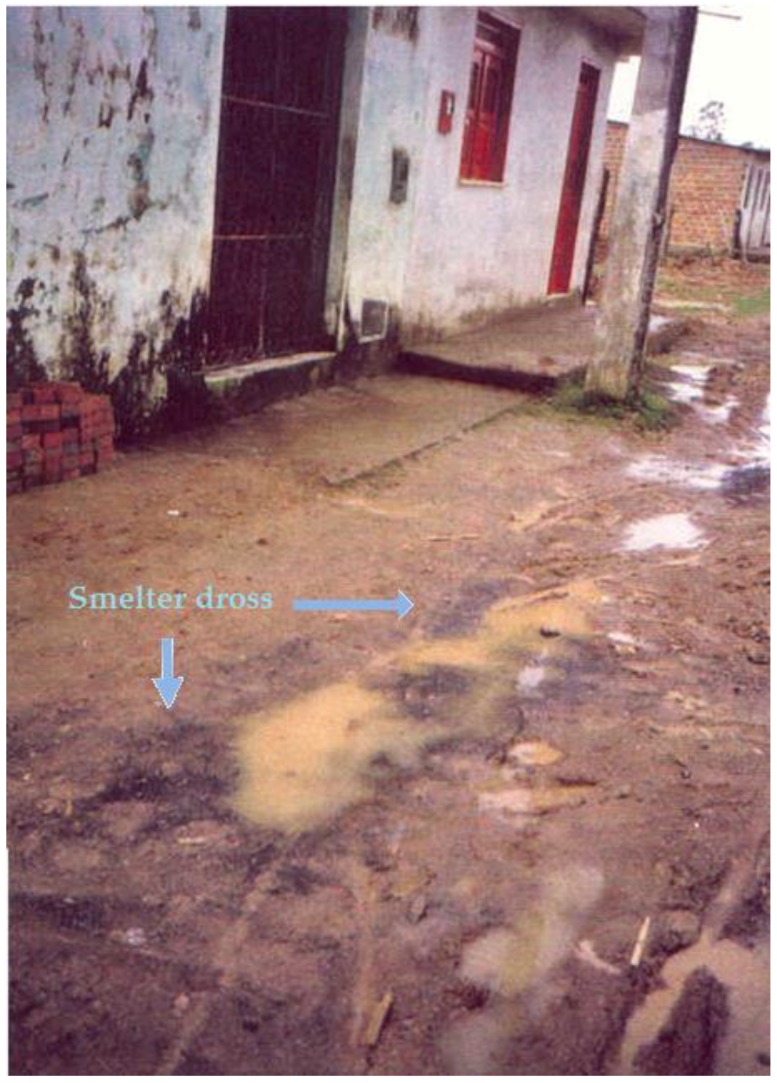
The dross polluting soil in front of households, 1998.

Since 1960, the population from Santo Amaro, State of Bahia, Brazil, has been exposed to gross environmental pollution caused by a primary lead smelter owned by the Peñarroya multinational group. The industrial residue obtained from smelted ore, the so-called “smelter dross”, was freely given to the population and the local Prefecture, and extensively used for paving households backyards, schoolyards, gardens, streets and other public places (Figure 1, Figure 2, Figure 3 and Figure 4) [1].

In 2002, the mean concentrations of chemical elements in the smelter dross were: PbO (4.06%, ZnO (9.47%), and Cd (57.3 mg/Kg) [2]. The dross, after granulated, was deposited on the grounds belonging to the smelter, without any cover or sealing, under the action of wind and rain [3]. The first studies on the contamination of the soils in the city of Santo Amaro were conducted in 1980 and comprised of 224 samples of surface soil of gardens, backyards and flower beds from households within 900 m from the smelter. The geometric mean of lead in the granulometry fraction of 200 mesh and lower (dry weight basis) was 4415 (geometric standard deviation (sd) 4.4) ppm, ranging from 32 to 107,268 ppm and the geometric mean of cadmium was 122 (geometric sd 3.2 ppm), ranging from 0.4 to 335 ppm. No other metals were determined. The attributed sources were mainly the presence of dross in the open, but also the atmospheric deposition of particles emitted from the smelter, both from the low chimney and fugitive emissions, as well as from the trucks transporting ore without proper cover and from the personal transport of the workers of the smelter to their homes. The State Government of Bahia demanded several measures from the smelter at this time, including the construction of a 90 m high chimney. A second study of soils was undertaken in 1985, with 87 surface soil samples collected and analyzed in the same manner. The values of lead and cadmium had decreased: the geometric mean of lead was 2529 (geometric sd 2.9 ppm), ranging from 236 to 83,532 ppm, a reduction of 42.9%; and the geometric mean of cadmium was 9.1 (geometric sd 3.5 ppm), ranging from 0.5 a 157 ppm, a reduction of 92.5 % [3].

The soil of this area is predominantly clay and silt with pH mean values around 6.6. The metal mobility in the soil can also be affected by the content of organic matter, clay, iron, and manganese. Fortunately, the pH values higher than 5 favors lead immobilization in clay and silt soils [4]. Lower pH values tend to increase bioavailability and mobility of most metals in soil [5]. Studies on the stability and leaching of some contaminants in the soil of Santo Amaro, especially Pb, Zn, and Cd, have been developed by different researchers during the last years [4,6,7]. However, there was a disagreement between the results of these researchers. No bioavailability studies have been conducted, but the levels of lead and cadmium in local vegetables and fruits collected within 1000 m radius from the smelter showed decreasing values with increasing distance from the smelter [8].

In 1980, the mean lead in the hair levels of 308 children from Santo Amaro increased consistently according to the content of lead in soil of their households [1]. An increase of 0.024 ppm in the mean Cd in the hair level of 225 children from Santo Amaro was estimated for each 1 ppm increase in the cadmium concentration in soil. [9]. In 1992, a cross-sectional study with 100 children aged 1–5 years who lived within 500 m from the smelter chimney reported that the mean zinc protoporphyrin levels were higher among those 11 children with visible dross around the household. Further, zinc protoporphyrin levels increased according to the number of times that the dross was used around the household in the last six years [10].

In 1989, the lead smelter was bought by a Brazilian group. In 1993, the smelter closed down, leaving 500,000 tons of contaminated dross in the industrial area and widespread in the urban area. There is no reliable estimate of the amounts of dross that was piled within the smelter grounds and that was brought to the urban area. Blood lead levels of 47 children aged 1 to 4 years old were 3.2 µg/100 mL higher among those with smelter dross visible at the household surroundings [11]. Young children are particularly vulnerable to the toxic effects of lead. There is no safe limit for lead concentration in the blood of children. A provisional, operational value of 5 µg/dL for blood lead concentration has been recommended for children [12]. In Santo Amaro, the mean blood lead level of children aged 1 to 9 years, living at less than 900 m from the smelter chimney, decreased from 59.1 ± 25.0 µg/dL to 36.9 ± 22.9 µg/dL, in 1985; and in 1998, among children aged 1–4 years, the mean blood level was 17.7 ± 7.3 µg/dL [10]. Because zinc protoporphyrin levels in the blood may denote toxic effects of lead on the haem synthesis, it was measured in 103 children aged 1 to 5 years, living at less than 500 m from the lead smelter chimney, in 1980, 1985, and 1992. Geometric means of zinc protoporphyrin levels were 132.8 µg/dL (geometric sd = 2.2) in 1980; 75.3 (geometric sd = 3.6) in 1985; and 65.5 (geometric sd = 1.7) in 1992 [11].

In spite of all the scientific evidence, the smelter management always stated that the dross was atoxic, based on the results of lixiviation tests made in laboratories accredited by State environmental agencies [13,14].

For almost 40 years, a large number of geochemical, geological, and epidemiological studies were conducted about the case of pollution by chemical elements in the Santo Amaro region. Until 2013, at least 110 items have been published: 36 articles, 40 technical reports, 5 Ph.D. theses, 15 MSc dissertations, 6 undergraduate monographies, and 8 others items [15].

Several studies have made technical diagnostics on the routes of lead contamination [4,14] and have proposed technical solutions to remediate the problems posed by the Santo Amaro smelter dross. In 2007, a firm proposed to recycle the Santo Amaro dross, aiming to profit with the lead and zinc amounts it contained. This initiative was moved by strictly economic reasons, but not for sanitary, social or ecological reasons. In 2016/2017, the prices of zinc [16] and lead [17] soared, reaching all-time highs. The recycling project was mainly directed to the exploration of the big piles of dross, left in the smelter grounds, at open air, estimated in 180,000 m^3^. Less attention was given to the amounts of smelter dross widely spread in the urban areas. A plant for processing the dross would use, only in the first three years, 45,000 tons of hydrochloric acid, a by-product to be provided by the Monsanto factory at Camaçari Hub, 62 km far from Santo Amaro. The initiative was firstly supported by the State Environmental Agency, with technical support of researchers from the School of Engineering of the Federal University of Bahia and from CETREL, the company responsible for water supplying, the treating and disposal of industrial debris, and the environmental monitoring of the Petrochemical Hub of Camaçari, State of Bahia [18]. In 2008, this proposal was refused by the State Environmental Council, reverting to a first approval by the State Environmental Agency after fierce political opposition from the representatives of the ecological movements [15].

A recent study considered the use of two techniques of soil remediation in Santo Amaro: soil washing using EDTA and soil thermal treatment at temperatures higher than 800 °C in an oxidant atmosphere. Costs with soil excavation, replacement, and stabilization of the soil were estimated at 20–30 million dollars [7].

None of the technical studies cited above took into account the perception of the people from Santo Amaro on the contamination of the soil and the solutions proposed to cope with this problem. Understanding the population perspective of contamination risks of health damages is an effective approach that integrates social elements that are not usually presented in epidemiological research. To know and understand the perception of the population in the contaminated area may allow for the prevention of diseases, education and more effective actions in health policies [19]. Recently, a study used a participatory methodology to involve citizens from Indianapolis (USA) in the collection and analysis of 2000 soil samples from approximately 500 households. This methodology led to the effective identification of metal concentration hotspots and the direct involvement of the local people in such a scale hardly met by using traditional epidemiological approaches [20].

This study aimed to present the local residents’ perception towards soil contamination by a lead smelter in Santo Amaro City, Brazil.

## 2. Materials and Methods

### 2.1. Study Design and Setting

A cross-sectional study on the perception of residents in the urban area of Santo Amaro City, Brazil, towards industrial dross hazards and proposals for its management was carried out. In 2010, Santo Amaro municipality had 57,800 inhabitants, 44,766 of them living in the urban areas. For hundreds of years until the first half of the 20th century, sugarcane has been the main crop in the area. From 1950 to 1990, the city experienced an ephemeral industrialization spurt. In 2010, among the employed aged (18 or older in the municipality), 9.5% worked in processing industries, 24.2% worked in agriculture, and 35.1% in the services sector. The Santo Amaro Municipal Human Development Index was 0.646, ranking 3186 among the 5565 Brazilian Municipalities; 45.7% of people aged 25 years or more had completed fundamental education (nine school years); mean per capita income was 392 reais (Brazilian currency) or US $218 [21].

### 2.2. Population Sampling

A map pinpointing the location of the 2042 poles of the electricity network of the urban area of Santo Amaro City was obtained from the State Electric Power Company. A simple random sample of 208 (sampling fraction = 10.8%) electricity poles was taken. The household closest to each of these poles was selected. Non-residential buildings or uninhabited households were substituted by the nearest one. A semi-structured questionnaire was applied to a resident aged 18-year or more who gave information about all residents in the household. Information was collected about family identification, occupational history, and the resident opinion dross-related hazards.

### 2.3. Residents’ Opinions

Questions on the resident’s opinion towards solutions proposed for the management of smelter dross from three different environmental compartments were collected: (a) in the households and its surroundings; (b) under street pavement and other public places; (c) and in the dross piles left in the closed smelter. The following seven independent (inclusive) answering options were suggested to the resident: (1) no opinion; (2) to leave the dross where it is; (3) to provide the residents with information about health risks related to the dross; (4) To remove the dross to another place, minimizing the residents exposure; (5) To coat the dross with other material, isolating it from the contact; (6) To reprocess the dross, making it nontoxic; (7) another solution. The resident’s opinion towards who should be liable for taking actions to manage the smelter dross was annotated, after the presentation of six independent (inclusive) answering options: (1) the residents of the households; (2) the local Prefecture; (3) the State Government; (4) the Federal Government; (5) the smelter enterprise; (6) other answer. A sample of typical smelter dross, packed into a transparent plastic bag, was presented to the resident. Subsequently, the resident was asked if he/she was able to recognize the smelter dross and on its use in the household, backyard, and surrounding (for paving sidewalks, streets, and other places). The questionnaires were applied by the research field team (four students of Medicine and four local residents from the local Association of the Victims Contaminated by Lead, Cadmium, Mercury and other Chemical Elements (AVICCA)), from February to March 2008.

### 2.4. Soil Sampling

Whenever the field research team investigator identified smelter dross in the surroundings of a selected household, he asked the resident’s permission to take a soil sample. A superficial soil sample was collected from the nearest place of the household, using a new stainless steel knife, and stored in a new labeled plastic bag.

### 2.5. Chemical Analysis

Each sample was homogenized with a porcelain mortar, lyophilized using a freeze dryer model Christ Alpha (Martin Christ Gefriertrocknungsanlagen GmbH, Osterode am Harz, Germany) with a vacuum pump from Vacuubrand GMBH, Wertheim, Germany, stored in a dark glass bottle and kept at temperatures between 18–23 °C until analysis. All material used was previously cleaned with Extran followed by 10% HNO_3_ for 24 hours and rinsed with MilliQ water. Approximately 0.500 g of each sample was digested in a microwave oven, with a concentrated acid mixture of fluoric acid (4 mL) and nitric acid 9 mL) in a high-pressure digestion vessel (DAK100) (Berghof, Eningen, Germany) according to the EPA 3052 procedure, with a ramp program totaling 59 minutes. The determination of the toxic metals was performed at the Pontifical Catholic University of Rio de Janeiro using ICP-MS (ELAN DRC II model, Perkin-Elmer Sciex, Norwalk, CT, USA) equipment, in the following conditions optimized by the Daily Performance method: 1250 W of radio frequency power and 1.0 L min^–1^ of nebulizing gas (Ar). The calibration was done with a multielement calibration solution containing 20 ug L^–1^ of each trace element. The Rh was employed as the internal standard with a concentration of 400 µg L^–1^. The blanks were prepared with nitric acid and the internal standard. The method was validated using two certificate reference samples of marine sediment, BCSS-1 e MESS-1. The accuracy was equal to or better than 90% in all cases. 

### 2.6. Statistical Analysis and Interpretation of Toxic Element Concentrations in the Soil

Values of the chemical element concentrations in soil were described by using basic statistical measures (median, mean, standard deviation and range). Pearson correlation coefficients were calculated for metal in soil concentrations after a logarithmic transformation. Mann-Whitney tests were used to compare the chemical element concentrations distributions according to the perception of the hazard (Yes/No groups, three questions) by residents in 39 households. Data were analyzed by using SPSS (Statistical Package for the Social Sciences), version 20 (IBM Corporation, Armonk, NY, USA).

Chemical element concentrations in the soil were classified according to soil quality guidelines proposed by the Brazilian Environmental National Council [22]. The Quality Reference Value (QRV) was the concentration of a determined substance in the soil that defines a soil as clean. The Prevention Value (PV) was the concentration of a determined substance in the soil above which hazardous alterations to soil quality can occur. This value indicates the quality of a soil that is able to sustain its primary functions, protecting the ecological receiving bodies. It must be used to discipline the introduction of substances into the soil and, whenever surpassed, the continuity of the activity should be submitted to another evaluation. The legal responsibilities for the introduction of the pollutant burdens should proceed with the monitoring of the resulting impacts. The Residential Investigation value (RIV) was the concentration of a determined substance in the soil above which there are direct or indirect risks to human health, considering a generic exposure scenario. The finding of a contaminant with a concentration above the RIV indicates the necessity of taking protective actions.

### 2.7. Ethical Aspects

The study was approved by the Ethical Review Board from Maternidade Climério de Oliveira, the Federal University of Bahia, protocol number 134/2007, respecting Brazilian Health National Council Resolution 466/12, as well as the Helsinki Declaration, 2008. All participants were informed and signed the consent form approved by the Ethical Board.

## 3. Results

Out of the 208 residents investigated, 143 (68.3%) stated that they were able to recognize the smelter dross in a typical sample; 31 (14.9%) reported its use in the household or in the backyard, and 100 (48.1%) stated its use in the household surroundings. In 23.6% of the households, some residents have already worked in the lead smelter, and in 17 (8.2%) households, someone planted, harvested, or had taken animals to graze on the smelter grounds after it closed down (Table 1).

Eighty-seven (41.8%) residents referred that the smelter dross was likely to cause harm and 56 (26.7%) reported that it was harming the health of one or more residents, at present (Table 2).

Only a small proportion (13.0%, 12.0%, and 10.1%, respectively) of the residents did not have an opinion about how to manage the smelter dross present in the three environmental settings: in the households and its surroundings; under street pavement and other public places; and in the dross piles left on the grounds of the closed smelter. Very frequently (38.0%, 36.1%, and 21.1%), the residents stated that they needed information about the health risks related to the dross as a solution for smelter dross management. The most frequent solution proposed, for dross in these three environmental settings, was “to remove the dross to another place, minimizing residents exposure” (27.9%, 34.1%, and 35.1%), reprocessing the dross, making it nontoxic appeared as the second option (15.9%, 21.1%, and 26.0%, respectively) (Table 3).

According to the residents, the smelter enterprise should be mainly responsible for taking cleanup actions of the smelter dross in the households and its surroundings (63.3%), under street pavement and other public places (60.1%), and the dross piles left in the closed smelter (62.0%). The local Prefecture was also frequently referred to as the putative responsible entity for taking actions concerning these three environmental settings: 51.9%, 60.1%, and 51.9%, respectively. In decreasing order of frequency, the State Government and the Federal Govern were also referred to as responsible entities (Table 4).

Table 5 shows the median, mean, standard deviation and maximum values for the concentrations of chemical elements in 39 soil samples. One sample presented the maximum values of lead, cadmium, zinc, and arsenic; and nickel ranked as the fourth highest concentration among the 39 soil samples, and it was excluded from the calculations of the Pearson correlation coefficients among the chemical elements.

Lead was strongly and significantly correlated (*p* < 0.05 or *p* < 0.001) with cadmium, zinc, and antimony (Table 6).

**Figure 5:**
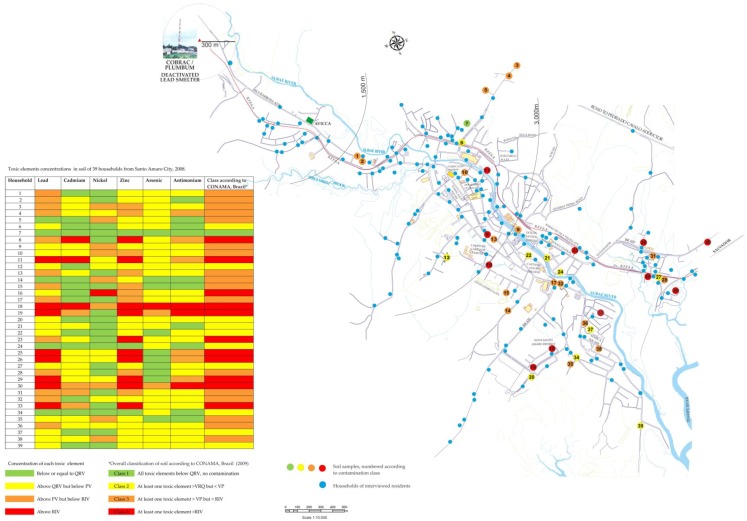
A map of Santo Amaro City showing the deactivated lead smelter, the 208 households investigated, including the 39 households from where soil samples were analyzed for chemical element concentrations and classified according to Brazilian quality standards, 2008.

Figure 5 shows a map of the Santo Amaro urban area that grew alongside Subaé River. The closed lead smelter appears in the upper left corner of the map. The blue circles denote the 208 households sampled for the interview with a resident. Green, yellow, orange and red circles, numbered 1 to 39, identify the location of the household where the sample was taken as well as its integrated classification taking in account all studied toxic elements according to Brazilian guidelines [22]: Class 1 means no contamination, Class 2 and 3 indicates that prevention and monitoring is required for at least one toxic element, and Class 4 indicates that actions such as investigation and intervention are required. The spots with concentrations of two or more chemicals elements exceeding the Prevention Value (PV) and/or the Residential Investigation Value (RIV) were widespread in the urban area. Considering the six toxic elements altogether, 11 (28.2%) out of the 39 households presented soils classified as class 4; 28 (71.8%) had soil concentrations exceeding PV and/or RIV; 17 (69.2%) belonged to Classes 2 or 3; and 1 (2.6%) household presented soil below or equal to the Quality Reference Value.

The concentrations in the soil were below the Quality Reference Value (QRV) for Lead (12.8%); Cadmium (41.0%); Nickel (53.8%); Zinc (7.7%); Arsenic (25.6%), and Antimony (26.6%). The Residential Investigation Value (RIV) was exceeded by Lead (23.1% of the samples), Cadmium (7.7%), Nickel (2.6%), Zinc (25.6%), Arsenic (2.6%), and Antimony (7.7%) (Table 7).

Out of the 39 residents in the households where the concentrations of chemical elements were determined in the soil, 30 (76.9%) stated that they were able to recognize the smelter dross; 12 (30.8%) referred to its use in his/her household or backyard; and 27 (69.2%) referred to its use in his/her household surroundings. The mean concentrations of lead, cadmium, nickel, zinc, arsenic or antimony in the soil did not differ significantly (at *p* < 0.10) according to the resident ability to recognize the smelter dross; reference to the use of smelter dross in his/her household or backyard; and reference to its use in his/her household surroundings (Table 8).

## 4. Discussion

This study confirms the results already described for Pb and Cd by previous studies about the gross contamination of the soil by smelter dross and its risks to the health of people from Santo Amaro [1,9,11]. In this study, where other toxic elements have been determined, the situation seems even worse. Looking at the levels found, not only are lead and cadmium are responsible for the risk to human health in this area, but also zinc, antimony, arsenic, and nickel. The integrated toxicity of all six elements can be expressed by the classification of the CONAMA [22] of each soil sample: 28.2% of the studied households presented soils were classified as class 4, meaning that action is considered necessary; 69.2% belonged to the CONAMA classes 2 and 3, when prevention measures such as monitoring and prevention are demanded, and only one household did not present contamination. Besides its toxicity, Pb is a probable carcinogenic element and nickel and arsenic in airborne particles have been proven to cause cancer in humans [23].

A recent study in Santo Amaro examined the soil of backyards from 230 households situated at less than 1.6 km far from the smelter chimney. The average lead concentration found was 1316 ppm; 80% of the samples were above 300 ppm (the CONAMA Residential Investigation Value), and 50% had values above 900 ppm [4]. These results confirm the important role of the smelter dross in the contamination scenario, especially for children. The average soil and dust ingestion rates for children are below 100 mg/day and most likely around 50 mg/day [24]; however, children with pica can ingest 25 to 60 g/day [25].

The strong correlation coefficients found between lead and other chemical element concentrations in soil confirm the common origin of, at least, a great part of these toxic pollutants: the dross produced by the lead smelter. In the past, the smelter dross has been widely distributed in the Santo Amaro urban space due to ignorance, negligence, or bad faith. Nowadays, whatever be the reasons for this social tragedy, people from the city (and surrounding areas) still ask for an urgent solution. Unfortunately, current technologies worldwide available to solve the problem are expensive, complex, time-consuming, and of questionable effectiveness [7,26,27].

The lead smelter had, and it still has, a close relationship with the citizens from Santo Amaro. In our sample, 23.6% out of the 208 respondents reported that someone in the household had already worked in the lead smelter and 8.2% planted, harvested, or had taken animals to graze on the smelter grounds after it closed down. Therefore, it is reasonable that residents from Santo Amaro frequently stated the fact that they were able to recognize the smelter dross: 68.3% out of the 208 respondents, and 76.9% out of the 39 residents in households where soil samples were taken. However, this ability was not strongly associated with the concentrations of toxic elements in the soil of their residences and surroundings (Table 8). These results reveal that the residents are unaware of the hazards posed by the contaminated soil. Therefore, the finding that 38.0% of the 208 respondents reported that the first solution for managing the dross found in their households was “to provide the residents with information about health risks related to the dross” makes sense.

The most frequent solution proposed for managing the dross found in the urban areas and in the smelter grounds was “to remove the dross to another place, minimizing the residents’ exposure”. This solution makes sense, particularly concerning the huge amounts of smelter dross left on the smelter grounds. The railway line that links Santo Amaro to Camaçari could transport the dross to CETREL, in the Petrochemical Hub of Camaçari. CETREL is a high-technology company, able to treat and recycle industrial debris.

According to the residents, the smelter enterprise was the main responsible entity for taking soil cleanup actions, closely followed by the local Prefecture. The State and Federal Government were less frequently held responsible. The dumping of smelter ore in streets and public places of Santo Amaro by local Prefecture trucks went on for many years and could be observed by the citizens. Further, because of the lead smelter’s great economic importance to the Municipality, the local Prefecture representatives always kept a very close political relationship with this industry.

The data from this study were collected in 2008, at a time when many solutions were proposed to clean up the pollution caused by the Santo Amaro lead smelter. The results of this study have been returned to each local resident. Technical reports were presented to the local Association of the Victims Contaminated by Lead, Cadmium, Mercury and other Chemical Elements (AVVICA), to the Prefecture, to the State Government representatives and to the State environmental agencies. After decades, the soil contamination problem persists, in spite of many technical discussions and political promises by the State and Federal Governments. Until now, no effective cleanup solution has been put into practice [13,15].

The empowerment of communities must play a prominent role in health promotion and environmental justice strategy. Empowered communities must set their priorities in health, make decisions and plan and implement strategies for their better health. The empowerment process represents more than just making decisions. It is a matter of autonomy, which requires the capacity of choice, knowledge, and the control of circumstances that may enhance vulnerabilities [28]. Among the Santo Amaro City residents aged 25 years or more, 45.7% had less than nine school years, 15% were illiterate, and only 4.0% had graduated from higher education; their mean per capita income was US $218 [21].

Although community, local leaders, neighborhood associations, State environmental agencies and governmental agencies were informed about our results, soil contamination is still a major environmental threat in the region. The majority (68.3%) of Santo Amaro residents stated that they were able to recognize the smelter dross. However, only 41.8% reported that the dross was likely to cause harm, and 10.1% reported that it caused no harm. This understanding reveals a lack of information about the present scientific knowledge on the matter. The community positioning may be explained by their complex, vulnerable situations involving a low income, a lack of basic infrastructure, a lack of environmental education, and a lack of political influence over government representatives. These affirmatives are reinforced by our findings that the majority of people from Santo Amaro City held the smelter enterprise liable for cleaning up the dross present in their households and surroundings (63.0%), followed by the local Prefecture (51.9%), State Government (43.8%) and Federal Government (36%). However, the community had no political way to warrant environmental protection actions. Governmental agencies should implement educational programs and preventive actions in the health and environment areas in order to protect these vulnerable populations [28]. Researchers also have the responsibility of empowering the communities affected by environmental damages and health disparities. Researchers should denounce environmental hazards to the Public Ministry and to other competent organs, leaving to them the responsibility of promoting interventions at the individual and collective level. Another researcher initiative could be the community-based participatory research, interacting with the communities, strengthening their autonomy, and involving people in the identification and solution of their environmental and health problems [29].

One strong point of this study was the participation of local residents as interviewers. They also belonged to the local Association of the Victims Contaminated by Lead, Cadmium, Mercury and other Chemical Elements. Together with the four medical students, this research team was capable of identifying households with visible smelter dross in their surroundings and to get reliable answers from local residents. However, in spite of their expertise, the research team members selected one household with no soil contamination and 10 households in class 2 soils, with concentrations above QRV and below PV. It is unlikely that these 11 soils were contaminated by smelter dross.

## 5. Conclusions

The quality of the urban soil of the Santo Amaro city is a matter of concern. There is unequivocal evidence that the dross produced by the lead smelter remains an important pollutant of the urban soil, representing a health risk to the population, especially to the children. The smelter dross is widely spread over the city. According to the residents, the smelter and the local Prefecture were most frequently held liable for taking soil cleanup actions. Residents requested information about the health risks related to the dross present in their households. The opinion of the Santo Amaro residents must be taken into account when solutions are proposed for problems of soil contamination in their city and in their households.

## Figures and Tables

**Table 1:** The occupational relationship with the lead smelter and with the smelter dross reported by residents in 208 households from Santo Amaro City, 2008.

Subject	*N*	%
Someone in the household has already worked in the lead smelter.	49	23.6
Someone in the household planted, harvested, or had taken animals to graze on the smelter grounds, after it closed down.	17	8.2
The respondent referred to be able to recognize the smelter dross in a typical sample.	143	68.3
Smelter dross used in the household or in the backyard.	31	14.9
Smelter dross used in the household surroundings (sidewalk, street, etc).	100	48.1

**Table 2:** The smelter dross ability to harm the health of people living in the household according to the opinion of 208 residents in Santo Amaro City, 2008.

Perception of Residents Regarding Possible Health Harm due to the Smelter Dross ^1^	*N*	%
It causes no harm	21	10.1
It is likely to cause harm	87	41.8
It has caused harm to one or more residents at some point	46	22.1
It is harming at present	56	26.7

^1^ Answers to four independent questions.

**Table 3:** The solutions proposed for smelter dross management present in the three different environmental settings according to the opinion of 208 residents in Santo Amaro City, 2008.

Proposed Solution	Smelter Gross in Households and Its Surroundings		Smelter Dross under Street Pavement and other Public Places		Smelter Dross Piles Left in the Closed Smelter	
	*N*	%	*N*	%	*N*	%
No opinion	27	13.0	25	12.0	21	10.1
To leave the dross where it is	33	15.9	54	26.0	15	7.2
To provide the residents with information about health risks related to the dross	79	38.0	75	36.1	44	21.1
To remove the dross to another place, minimizing residents exposure	58	27.9	71	34.1	73	35.1
To coat the dross with other material, isolating it from the contact	29	13.9	35	16.8	48	23.1
To reprocess the dross, making it non-toxic	33	15.9	44	21.1	54	26.0
Another solution	6	2.9	2	1.0	4	1.9

**Table 4:** The putative responsible entity for taking actions to manage the smelter dross according to the opinion of 208 residents in Santo Amaro City, 2008.

Putative Responsible	Smelter Dross in the Households and Their Surroundings		Smelter Dross under Street Pavement and other Public Places		Smelter Dross Piles Left in the Area of the Closed Smelter	
	*N*	%	*N*	%	*N*	%
The residents of the households	6	2.9	4	1.9	4	1.9
Local Prefecture	108	51.9	125	60.1	108	51.9
State Govern	91	43.8	87	41.8	98	47.1
Federal Govern	75	36.1	77	37.0	83	39.9
The smelter enterprise	131	63.0	125	60.1	129	62.0
Other	2	1.0	2	1.0	2	1.0

**Table 5:** The statistical characteristics of toxic elements concentrations (ppm) in the soils of 39 households and surroundings from Santo Amaro City, 2008.

Chemical Element	Median	Arithmetic Mean	Standard Deviation	Minimum	Maximum ^1^
Lead	77	1040	3162	5.9	17,862
Cadmium	0.58	2.73	9.9	0.06	61.6
Nickel	11.8	22.0	23.2	3.1	101.0
Zinc	295	3484	10,835	30	62,084
Arsenic	5.2	36.3	187.4	0.0	1176.0
Antimony	0.8	21.5	122.4	0.2	766.0

^1^ One soil sample presented all these maximum values, except for nickel, that ranked as the fourth highest concentration.

**Table 6:** The Pearson correlation coefficients matrix for chemical element concentrations (after logarithmic transformations) in soils of 38 households and surroundings from Santo Amaro City, 2008.

	Cadmium	Nickel	Zinc	Arsenic	Antimony
Lead	0.826 **	0.230	0.961 ***	0.244	0.800 ***
Cadmium	-	0.220	0.791 ***	0.395 *	0.795 ***
Nickel		-	0.319*	−0.007	0.282
Zinc	-	-	-	0.195	0.808 ***
Arsenic	-	-	-	-	0.415 **

* *p* < 0.05; ** *p* < 0.01; *** *p* < 0.001.

**Table 7:** The quality of soil indicators according to the Brazilian guidelines for soils in the 39 households from Santo Amaro City, 2008.

Chemical Element	QRV ^1^ (ppm)	Below QRV		PV ^2^ (ppm)	Above QRV but Below PV		RIV ^3^ (ppm)	Above PV but Below RIV		Above RIV	
		*N*	%		*N*	%		*N*	%	*N*	%
Lead	17	5	12.8	72	13	33.3	300	12	30.8	9	23.1
Cadmium	<0.5	16	41.0	1.3	16	41.0	8	4	10.3	3	7.7
Nickel	13	21	53.8	30	8	20.5	100	9	23.1	1	2.6
Zinc	60	3	7.7	300	17	43.6	1000	9	23.1	10	25.6
Arsenic	3.5	10	25.6	15	26	66.7	55	2	5.1	1	2.6
Antimony	<0.5	10	25.6	2	20	51.3	10	6	15.4	3	7.7

^1^ QRV—Quality Reference Value; ^2^ Prevention Value; ^3^ Residential Investigation Value. (CONAMA, 2009) [22].

**Table 8:** The concentrations (ppm) of chemical elements in the soil according to the perception of the hazard by residents in the 39 households from Santo Amaro City, 2008.

Chemical Element	Measure ^1^	Able to Recognize the Dross in a Typical Sample		Smelter Dross Used in the Household or in the Backyard		Smelter Dross Used in the Household Surroundings (Sidewalk, Street, etc)	
		Yes (30)	No (9)	Yes (12)	No (27)	Yes (27)	No (12)
Lead	Mean	594	2526	361	1340	1397	234
	Median	76	228	81	75	81	64
	Max.	8800	17,862	3136	17,862	17,862	1128
Cadmium	Mean	1.08	8.21	1.33	3.34	3.64	0.67
	Median	0.59	0.50	0.50	0.60	0.62	0.44
	Max.	10.60	61.60	9.40	61.60	61.60	2.40
Nickel	Mean	20.8	26.9	11.6	26.6	21.3	23.6
	Median	9.9	20.0	8.1	14.5	11.8	11.3
	Max.	101.0	60.7	30.9	101.0	73.4	101.0
Zinc	Mean	2075	8179	694	4723	4605	961
	Median	266	412	183	412	296	313
	Max.	29,182	62,084	3986	62,084	62,084	4795
Arsenic	Mean	6.8	134.8	7.7	49.0	49.1	7.5
	Median	5.4	4.9	4.9	5.3	5.3	4.4
	Max.	39.8	1176.0	39.8	1176.0	1176.0	39.8
Antimony	Mean	2.2	86.1	2.1	30.2	30.0	2.4
	Median	0.8	1.3	0.6	1.1	0.8	1.2
	Max.	15.5	766.0	15.1	766.0	766.0	15.1

^1^ No statistically significant differences were found (at *p* < 0.10, Mann–Whitney tests) between chemical element distributions according to the Yes/No groups.
